# Long non-coding RNAs (lncRNAs) in spermatogenesis and male infertility

**DOI:** 10.1186/s12958-020-00660-6

**Published:** 2020-10-30

**Authors:** Meghali Joshi, Singh Rajender

**Affiliations:** Division of Endocrinology, Central Drug Research Institute, Lucknow, UP India

**Keywords:** Long non-coding RNA, lncRNA, lncRNA regulation, lncRNA databases, Transcriptome, Spermatogenesis, Male infertility

## Abstract

**Background:**

Long non-coding RNAs (lncRNAs) have a size of more than 200 bp and are known to regulate a host of crucial cellular processes like proliferation, differentiation and apoptosis by regulating gene expression. While small noncoding RNAs (ncRNAs) such as miRNAs, siRNAs, Piwi-interacting RNAs have been extensively studied in male germ cell development, the role of lncRNAs in spermatogenesis remains largely unknown.

**Objective:**

In this article, we have reviewed the biology and role of lncRNAs in spermatogenesis along with the tools available for data analysis.

**Results and conclusions:**

Till date, three microarray and four RNA-seq studies have been undertaken to identify lncRNAs in mouse testes or germ cells. These studies were done on pre-natal, post-natal, adult testis, and different germ cells to identify lncRNAs regulating spermatogenesis. In case of humans, five RNA-seq studies on different germ cell populations, including two on sperm, were undertaken. We compared three studies on human germ cells to identify common lncRNAs and found 15 lncRNAs (LINC00635, LINC00521, LINC00174, LINC00654, LINC00710, LINC00226, LINC00326, LINC00494, LINC00535, LINC00616, LINC00662, LINC00668, LINC00467, LINC00608, and LINC00658) to show consistent differential expression across these studies. Some of the targets of these lncRNAs included CENPB, FAM98B, GOLGA6 family, RPGR, TPM2, GNB5, KCNQ10T1, TAZ, LIN28A, CDKN2B, CDKN2A, CDKN1A, CDKN1B, CDKN1C, EZH2, SUZ12, VEGFA genes. A lone study on human male infertility identified 9879 differentially expressed lncRNAs with three (lnc32058, lnc09522, and lnc98497) of them showing specific and high expression in immotile sperm in comparison to normal motile sperm. A few lncRNAs (Mrhl, Drm, Spga-lncRNAs, NLC1-C, HongrES2, Tsx, LncRNA-tcam1, Tug1, Tesra, AK015322, Gm2044, and LncRNA033862) have been functionally validated for their roles in spermatogenesis. Apart from rodents and humans, studies on sheep and bull have also identified lncRNAs potentially important for spermatogenesis. A number of these non-coding RNAs are strong candidates for further research on their roles in spermatogenesis.

## Background

**Fig. 1:**
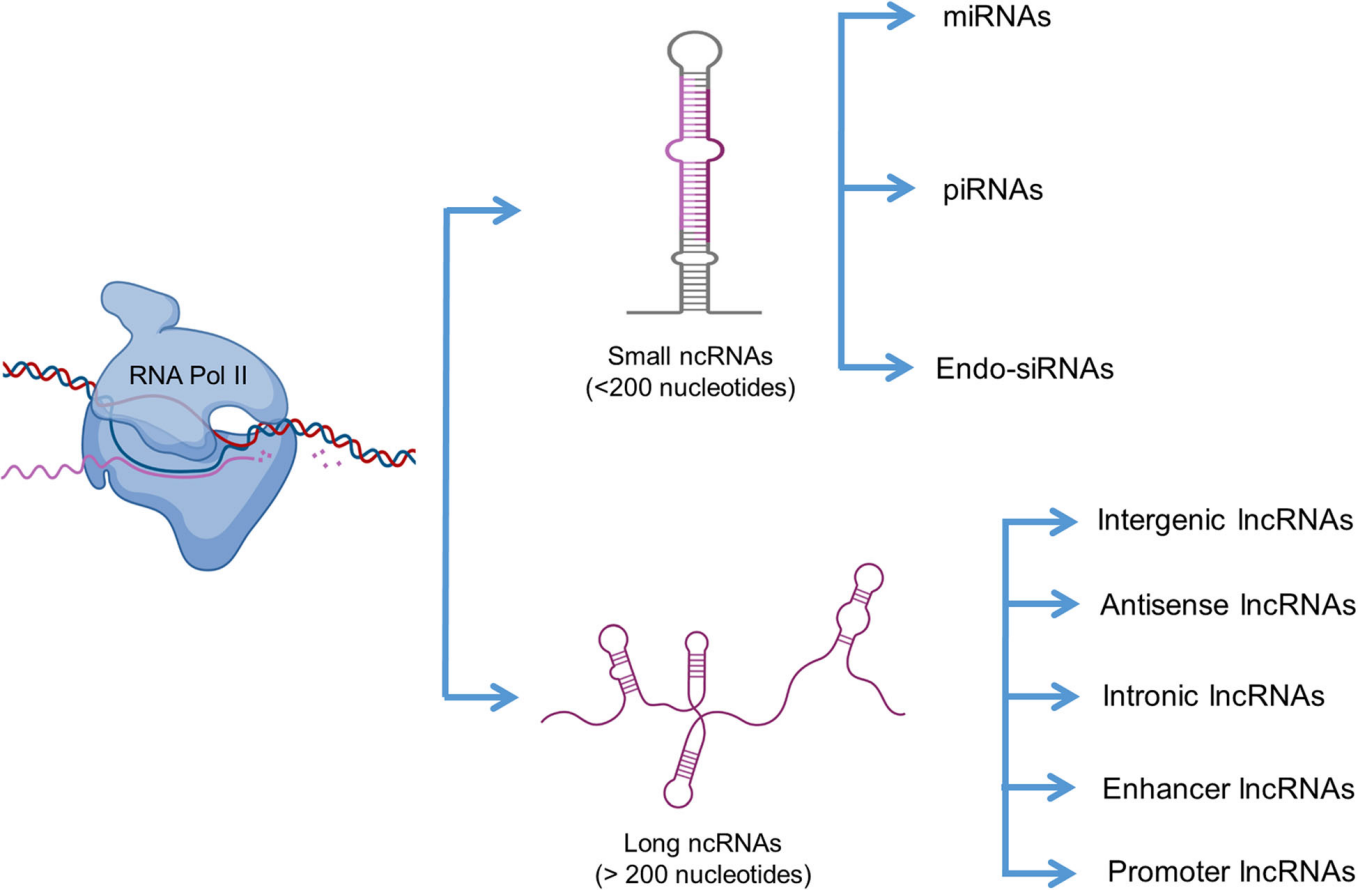
Classification of non-coding RNAs. Based on size non-coding RNAs can be divided into small non-coding RNAs and long non-coding RNAs

According to the central dogma, DNA is transcribed into mRNA, which is translated into protein, and the latter perform most of the functional, structural, and regulatory roles in various cellular processes. Nevertheless, it was found that protein coding genes in both lower and higher organisms are similar and are not enough to account for the complexity of higher organisms, hence challenging the protein centric view. With the advent of high-throughput technology, it was found that the majority of the genome in humans is transcribed and out numbers the current gene annotations. Moreover, a majority of these transcripts do not get translated into proteins. These transcripts which are not translated into proteins are known as the noncoding RNAs (ncRNAs) [1, 2]. Earlier, ncRNAs were considered as ‘junk’, but we realized the importance of this junk chunk only recently [3]. Non-coding RNAs can be classified on the basis of their size. Non-coding RNAs having size less than 200 bp are called small non-coding RNAs (sncRNAs) and those above 200 bp are called long non-coding RNAs (lncRNAs) [4] . MicroRNA (miRNA), small interfering RNAs (siRNAs) and Piwi-interacting RNA (piRNA) come under the category of sncRNAs (Fig. 1). Several studies have documented the role of small non-coding RNAs in male germ cell development [5, 6]. Research on lncRNAs has picked up the pace only recently with reports on the role of lncRNAs in regulating important genes in human and rodent embryonic stem cell (ESC) models [7, 8]. LncRNAs perform a wide variety of functions in mammals; for instance, they regulate the genes involved in chromatin modification, inactivate the X-chromosome by direct binding, inhibit antisense mRNA expression, promote antisense mRNA degradation, act as competing endogenous RNA (ceRNAs) to inhibit the function of miRNAs and bind to the DNA binding proteins to inhibit their interaction with the target genes [9].

Long non-coding RNAs have been reported to regulate a variety of cellular processes such as differentiation, proliferation and apoptosis. The differential expression of lncRNAs in human diseases highlights their roles in various biological processes. It has been reported that three lncRNAs: Peril, Evf2 (Dlx6os1) and linc-Brn1b are important for neural development [10, 11]. Several lncRNAs play important role in the development of organs during embryogenesis, such as lncRNA-Fendrr, which is transcribed divergently from the transcription factor Foxf1. The mouse knock-out model of Fendrr was seen to have defects in lungs, heart and gastrointestinal tract [11]. Further, it was found that at the embryonic stage E13.5, the developing lungs of the KO mouse had smaller size with globular and disorganised lobes. These KO mice die soon after birth due to breathing problems [12]. Several lncRNAs are reported to play crucial role in stem cell maintenance and differentiation. For example, RMST [13], Miat [14], Tunar [15] are reported to have roles in neural differentiation. LncRNAs like Braveheart [16], Alien [17], SENCR [18] and hoxBlinc [19] are required for cardiac, endoderm, endothelial and hematopoietic differentiation, respectively. A large number of lncRNAs are involved in a variety of cancers, such as ANRIL [20], CARLo-5 [21], *FAL1* [22], *GAS5* [23], HOTAIR [24], HOTTIP [25], PCGEM1 [26], PCA3 [27]. The diversity of lncRNAs and their biological functions are taking giant leap at present.

Till date many potential lncRNAs have been reported in spermatogenesis and male infertility, however, very few of them have been validated and functionally characterized. A comparison of the findings across these studies can lead to the identification of lncRNAs important for spermatogenesis which could also be exploited as markers of male infertility. Therefore, in this article, we have reviewed the long noncoding RNAs (lncRNAs), their genomic locations, databases for lncRNA prediction and analysis, recent discoveries made with respect to spermatogenesis and male fertility, and discuss important lncRNAs known to play a functional role in spermatogenesis and male fertility.

### Long non-coding (lnc) RNAs

LncRNAs (long non-coding RNAs) are defined as the non-protein-coding RNAs, which are at least 200 nucleotides in length [28, 29]. Like mRNAs, lncRNAs are transcribed by RNA polymerase II and sometimes processed like mRNAs i.e. have 5mG cap, spliced and polyadenylated [30]. With the advent of sensitive, high throughput technologies like next-generation sequencing (NGS) and microarray, several lncRNAs have been discovered, but till date only few have been functionally characterized by gene specific studies.

### Genomic contexts of lncRNAs

**Fig. 2:**
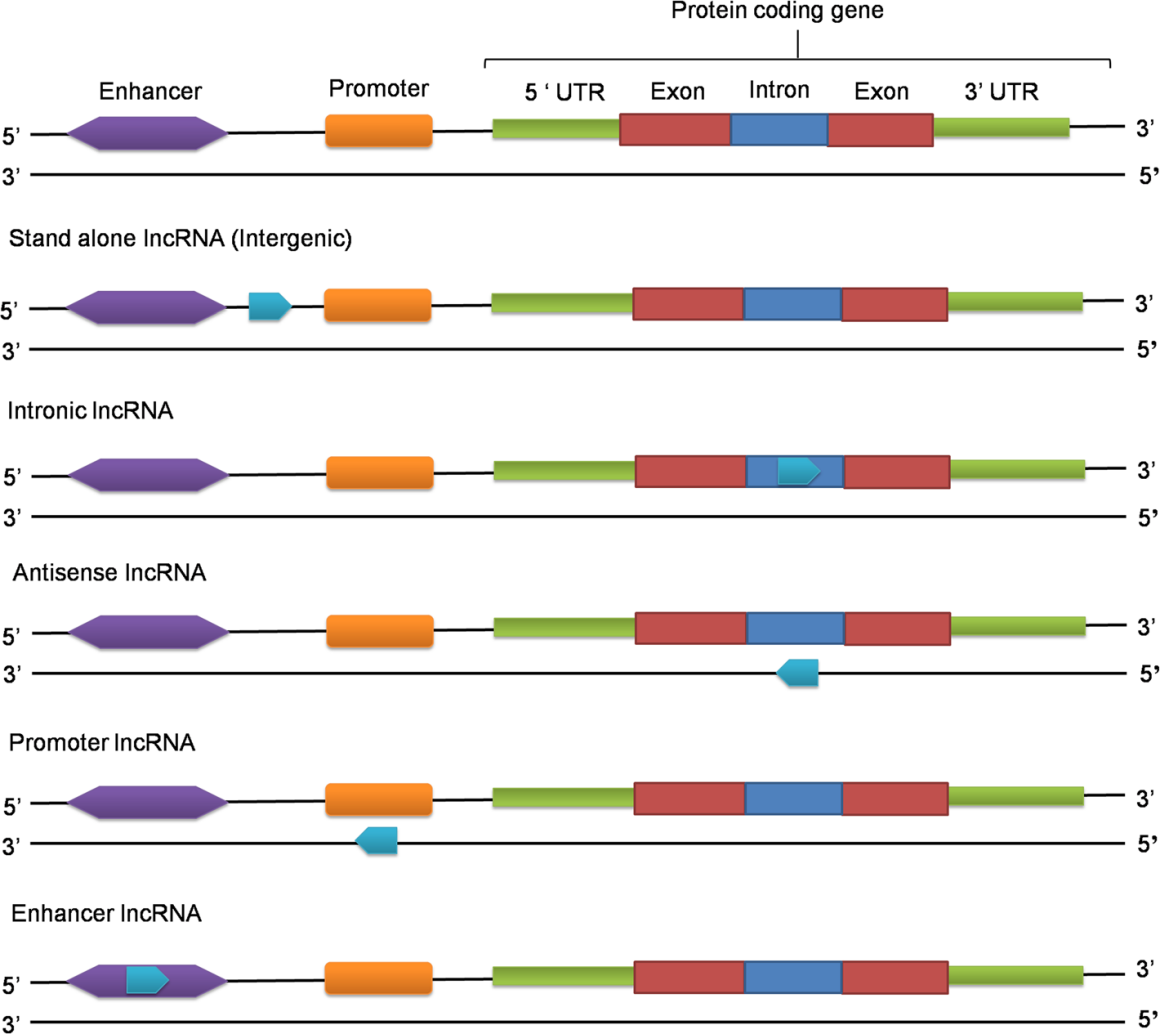
Genomic contexts of lncRNAs. LncRNAs (blue) may be stand-alone transcription units, intronic, antisense to other genes, or transcribed from promoters or enhancers

LncRNAs can be classified on the basis of their genomic location i.e. depending upon where from these RNAs are transcribed. These can be grouped into five broad classes: stand-alone lncRNAs, natural antisense transcripts, pseudogenes, long intronic ncRNAs, promoter-associated transcripts, and enhancer transcripts (Fig. 2).

#### Stand-alone lncRNAs

Stand-alone lncRNAs (also known as lincRNAs or large intergenic noncoding RNAs) are transcribed from a distinct genomic loci and do not overlap with protein-coding genes [31–33]. They have an average length of 1 kb and are transcribed by RNA Pol II. These transcripts are usually polyadenylated and spliced. In humans, more than half of the lncRNAs constitute lincRNAs [34]. LincRNAs have been reported to have functional roles in a wide-variety of species. Till date, these lncRNAs have been reported in human, mouse, rat, pig, bovine, bull, zebrafish, *C. elegans*, yeast, *Arabidopsis thaliana* [34–37]. Xist [38], HOTAIR [39], H19 [40] and MALAT1 [41] are some known examples of stand-alone lncRNAs.

#### Natural antisense transcripts (NATs)

Natural antisense transcripts are transcribed from the opposite sense strand [41, 42]. Generally, NATs are transcribed from both the ends of sense genes and are complementary to the 5′ and 3′ ends of sense mRNAs [42–44]. As NATs are complementary to the protein–coding mRNAs, these regulate gene expression by RNA: RNA interaction. The best example is the X- inactive-specific transcript Xist/Tsix transcript pair, which is involved in X-chromosome silencing [45]. Just like lincRNAs, this class of lncRNA has been reported in a wide variety of species, including humans, mouse, rat, cattle, chicken, pigs and yeast [35, 37, 46, 47].

#### Pseudogenes

Pseudogenes are ‘relics’ of genes, which are non-functional and do not encode for any protein [46]. They are the result of duplication of a parent gene or retrotransposition of the mRNA sequence into a different genomic locus. These are non-functional as a consequence of non-sense, frameshift and other mutations. Approximately, 2–20% of the pseudogenes are transcribed into RNAs, which come under this category of lncRNAs. Pseudogenes derived lncRNAs have been reported in both human and mouse. Lethe, a pseudogene derived lncRNA, was induced by pro-inflammatory cytokines via TNFα signaling in mouse [47]. Pseudogenes derived lncRNAs have also been reported to be critical for cancer development and progression. For instance, DUXAP10 was found to be up-regulated in colorectal cancer [48]. Many studies have revealed that pseudogenes regulate gene expression of their parental gene by producing natural siRNAs or antisense transcripts [49].

#### Long intronic ncRNAs

It has been known that introns harbour small ncRNAs, such as snoRNAs and miRNAs. Several transcriptome studies have found that many long transcripts are encoded by the introns of annotated genes [50, 51]. Till date only few have been studied, but it has been seen that these are dysregulated in cancer and many have differential expression pattern in disease conditions [52]. The intronic lncRNAs have been reported in human, mouse, rat, cattle, chicken, pig and *Arabidopsis* [35, 37, 53, 54]. An lncRNA, ANRASSF1, epigenetically regulates the expression of a tumor-suppressor gene, RASSF1, in tumorigenesis [53]. Similarly, an lncRNA SYISL, which is highly expressed in muscles in mouse, has been reported to recruit epigenetic machinery (EZH2, PRC2) to the promoters of p21, MyoG, MCK and Myh4, leading to epigenetic silencing [54] . In *Arabidopsis thaliana*, COLDAIR, an intron present in the FLC (flowering repressor locus) gene, is responsible for plant vernalization [55].

#### Promoter-associated long transcripts and enhancer RNAs

Promoter-associated long RNAs (PALPs) and enhancer-associated RNAs (eRNAs) are lncRNAs, which are transcribed from promoters and enhancers by RNA Pol II [56]. Both promoter and enhancer-associated lncRNAs are known in human and mouse [57, 58]. Promoter associated RNAs are transcribed from the transcription start sites (TSS) of protein coding or non-coding genes. These are cis-acting elements which regulate the expression of their neighbouring genes. The PALPs have recently emerged as an important epigenetic regulator of genes involved in a variety of diseases [59]. Myoparr is a promoter associated lncRNAs, which is transcribed from the promoter region of human and mouse myogenin gene. It activates the expression of myogenin gene by interacting with Ddx17 and PCAF and inhibits proliferation by activating myogenic microRNA expression, thereby promoting myogenesis [57]. Enhancer associated RNAs (eRNAs) are approximately 2 kb long and are generally non-polyadenylated RNAs [60]. It has been reported that eRNAs are involved in chromatin opening and promoting RNA Pol II binding at the transcription site of the target gene [61, 62]. An enhancer associated RNA, CARMEN, was found to be highly expressed in human cardiac precursor cells (CPC) where it interacts with SUZ12 and EZH2 (components of PRC2 complex) and promotes cardiac specification and differentiation in CPC [58].

### Regulation by long non-coding RNAs

**Fig. 3:**
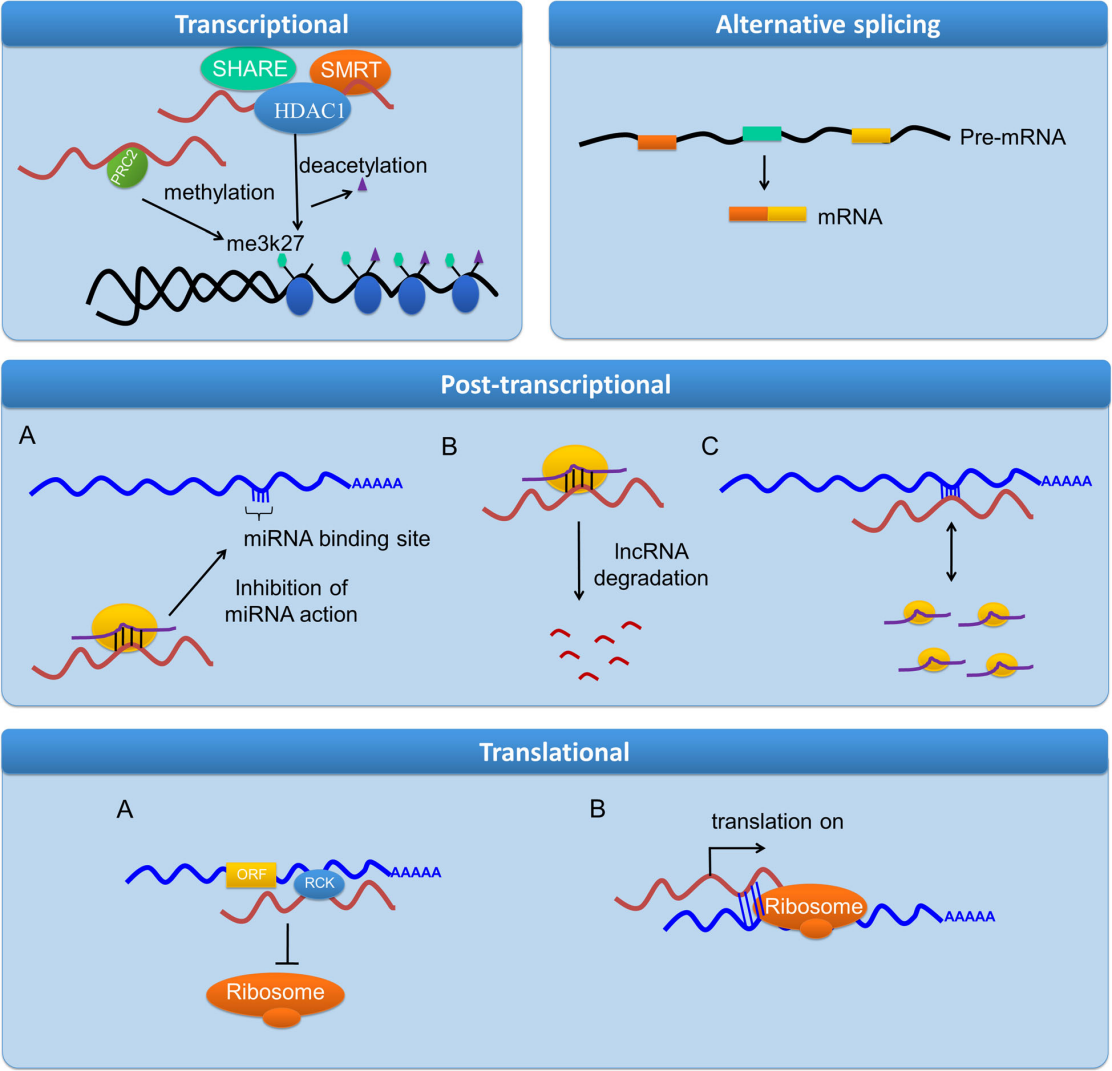
Gene expression is regulated by lncRNAs in three ways. Transcriptional: lncRNA interacts with methylation and de-acetylation complex to affect gene expression. Post-transcriptional: lncRNA interacts with miRNA to inhibit its action (**a**), lncRNA is degraded by miRNA (**b**), competitive binding of lncRNA and miRNA to the target site on mRNA (**c**). Affecting alternative splicing: lncRNA facilitates alternative splicing by binding to the target site on pre-mRNA. Translational regulation: lnc recruits translational repressor to block translation (**a**), lnc recruits polysomes to promote translation (**b**)

Gene regulation is a complex process, which takes place at transcriptional, post-transcriptional and translational levels (Fig. 3). Long non-coding RNAs in association with other molecules regulate gene expression in the following ways:

#### Transcriptional level

LncRNAs are involved in epigenetic gene regulation and genomic imprinting, such as X-inactive specific transcript (XIST), AIR, H19 [63]. In females, during early embryonic stage one X-chromosome is inactivated to ensure dosage compensation with regard to hemizygous males [64]. This inactivation of X-chromosome is initiated by the recruitment of PRC2 (polycomb repressive 2) complex to specific sites, resulting in H3k27me3 (Histone H3 lysine K27 trimethylation). H19, Airn, Kcnq1ot1, Meg3, and Meg8 are the examples of lncRNAs that are involved in controlling genomic imprinting [65]. In this process, gene expression is regulated by the recruitment of DNA and histone methyl transferases for DNA methylation and histone modifications, respectively [66]. This type of regulation has been reported in human, mouse and yeast [66, 67].

#### Post-transcriptional level

After transcription, long non-coding RNAs can regulate the expression of miRNAs by working as a miRNA sponge. It can also interfere with the function of miRNA by competing for its target site on mRNA. Moreover, miRNA can also regulate the expression of lncRNAs by binding to its 3’UTR region. This type of regulation has been reported in human, mouse and rat [68–71].

A. There are several non-coding RNAs, which regulate gene expression by binding to the target mRNA through sequence complementarity. There are certain lncRNAs which work as competitive endogenous RNAs (ceRNAs), also called miRNA sponges because they contain binding sequences for miRNAs [72]. These ceRNAs interact with miRNAs and impair the interaction between miRNA and its target mRNA. For example, lnc-MG blocks miR-125b expression and controls IGF2 (Insulin growth factor 2) levels [73].
B. MicroRNAs can also regulate the expression of lncRNAs [68]. For example, miR-101 and miR-217 negatively regulate lncRNA MALAT1 (metastasis associated with lung adeno-carcinoma transcript1) [69] and miR-449a blocks lncRNA NEAT1 (nuclear enriched abundant transcript 1) expression in cancer [74].
C. Long non-coding RNAs can interrupt in miRNA function by competing for its target site on mRNA. A very good example of this is the binding of lncRNA BACE1-AS (β-site amyloid precursor protein cleaving enzyme 1- antisense transcript) to its sense partner, where miR-485-5p binds, thus impairing the function of miR-485-5p in Alzheimer’s disease [75].

#### Alternative splicing

The process of alternative splicing takes place in both animals and plants to generate different mRNA isoforms [76]. It was recently found that lncRNAs regulate the process of alternative splicing by interacting with the splicing factors or by forming RNA-RNA duplexes with pre-mRNAs [70]. Till date, this type of regulation by lncRNA has been reported in human and mouse [77]. Recently, it has been reported that lncRNAs NEAT1 and MALAT1 co-localize with nuclear speckles containing the splicing factor SC35, suggesting their involvement in the process of alternative splicing [71].

#### Translation

A. LncRNAs play important roles in regulating the translation process as well. LncRNAs regulating target genes at the translational level have been reported in human and mouse [68]. LncRNA-p21 was reported to suppress the translation of JunB and β-catenin mRNA by recruiting translational repressors [68].
B. The translation of Uchl1 (ubiquitin carboxyl-terminal hydrolase L1) is facilitated by the lncRNA generated from its antisense strand AS-Uchl1, which recruits polysomes with the help of a repetitive domain SINEB2 to promote a cap- independent translation [68].

#### Post-translational modifications

LncRNAs can also modulate the post-translation modifications (PTM) of a protein by masking the PTM sites, which has been reported in both human and mouse [78, 79]. Lnc-DM is a cytosolic lncRNA exclusively expressed in human conventional dendritic cells (DCs). This lncRNA regulates the differentiation of dendritic cells by phosphorylating STAT3 on Tyr205, thereby preventing the binding of the protein tyrosine phosphatase SHP1 [80]. Similarly, a NF-κB interacting lncRNA NKILA interacts with IκB and inhibits its phosphorylation, leading to the activation of NF-κB and suppression of breast cancer metastasis [81].

### Databases for lncRNA prediction and analysis

High throughput studies, such as, microarray and deep sequencing, have identified a huge number of non-coding RNAs [77]. Around 90,000 lncRNAs have been reported by high throughput transcriptome studies [78], but only 16,000 are annotated in GENCODE [79]. Many new lncRNA databases have been developed to annotate such a large number of lncRNAs (Table 1). The number of lncRNAs stored in the databases ranges from < 2000 to > 70,000 transcripts, Noncode v3.0 being the largest database, storing > 73,000 transcripts. DIANA-LncRNA is the database which contains the largest number of computationally predicted lncRNAs (> 56,000 transcripts) as well as the largest number of experimentally validated lncRNAs (2958 transcripts). These databases automatically generate results as lists or tables according to the query. Additionally, most of them also provide graphical visualizations as diagrams or plots (Noncode v3.0, DIANA-LncBase, CHIPBase, LNCipedia, and lncRNome) [96]. There is at least one database which predicts the targets for lncRNAs [94]. This database predicts lncRNA-lncRNA and lncRNA-mRNA interaction in human and mouse. Another database enlists targets for lncRNA in disease-specific conditions, such as cancer, atherosclerosis, cardiac fibrosis and pulmonary hypertension [95]. The information regarding stored lncRNAs and their biological annotations are taken from the literature, computational predictions or primary data repositories. GENCODE project, which is a part of the ENCODE project [97] is a primary data repository providing accurate annotations of human genome, including non-coding RNAs [98]. On the other hand, database like lncRNADisease and functional lncRNA databases rely on manually identified, literature extracted annotations.

**Table 1:** List of databases for lncRNA annotation and characterization

Database	Description	Organism	Website	Reference
CHIPBase v2.0	It explores the transcriptional regulatory networks of non-coding RNAs (ncRNAs) and protein coding genes (PCGs).	Human and mouse	Rna.sysu.edu.cn/chipbase/	[82]
DIANA-LncBase	This database contains information for both predicted and experimentally verified miRNA-lncRNA interactions.	Human and mouse	Diana.imis.athena-innovation.gr	[83]
LNCipedia	It is a public database for long non-coding RNA (lncRNA) sequences and annotation. It contains 127,802 transcripts and 56,946 genes.	Human	www.lncipedia.org	[84]
lncRNAdb v2.0	This database provides a comprehensive annotation of eukaryotic long non-coding RNAs.	71 different organisms	www.lncrnadb.org	[85]
lncRNADisease	Database containing experimentally validated lncRNAs-disease associations.	Human	Cmbi.bjmu.edu.cn/lncrnadisease	[86]
lncRNome	Database containing comprehensive list of lncRNA in humans. It contains information for over 17,000 long non-coding RNAs in humans.	Human	Genome.igib.res.in/lncRNom	[87]
Noncode v5.0	Integrated knowledge database of non-coding RNAs containing non-coding RNAs expressing in 17 different species.	Human, mouse, rat, chicken, fruit fly, zebrafish, *C. elegans*, yeast, *Arabidopsis*, chimpanzee, gorilla, orangutan, rhesus macaque, opossum platypus and pig	www.bioinfo.org/noncode/	[88]
The Functional lncRNA database	Database having information of long non-protein-coding transcripts that have been experimentally shown to be both non-coding and functional. Currently the database contains lncRNAs from Human, Mouse and Rat.	Human, mouse and rat	www.valadkhanlab.org	[89]
Encyclopedia of DNA Elements (ENCODE) project	It contains comprehensive list of functional elements in the human genome, including elements that act at the protein and RNA levels.	Human	www.encode.project.org	[90]
StarBase v3.0	Contains information of more than 1.1 million miRNA-ncRNA, 2.5 million miRNA-mRNA, 2.1 million RBP-RNA and 1.5 million RNA-RNA interactions from sequencing data.	Human, mouse and *C. elegans*	www.starbase.sysu.edu.cn	[91]
RNA-Protein Interaction Prediction (RPI Seq)	This database helps in predicting any possible interaction between lncRNA of known sequence and proteins.	Human, mouse, yeast, fruit fly and *E. coli*	Prisdb.gdcb.iastate.edu/RPISeq/	[92]
Lnc2Catlas	It gives information about lncRNAs and their association with cancer risk. It provides association between 33 different cancers and 27,670 lncRNA transcripts.	Human	Lnc2catlas.bioinfotech.org	[93]
LncRIsearch	It is a web server which predicts human and mouse lncRNA-lncRNA and lncRNA-mRNA interactions. It uses RI blast to predict RNA-RNA interaction. It also provides information of tissue-specific expression and subcellular localization data for the lncRNAs.	Human and mouse	rtools.cbrc.jp/LncRRIsearch/	[94]
LncTarD	It provides information about disease- related lncRNAs for humans. This database provides a comprehensive resource of 2822 key lncRNA-target regulations, lncRNA influenced functions, and lncRNA-mediated regulatory mechanisms in human diseases.	Human	Biocc.hrbmu.edu.cn/LncTarD/	[95]

All the databases provide information about lncRNAs identified in humans. Databases which provide information of mouse lncRNAs are DIANA-LncBase, CHIPBase, lncRNAdb, Noncode v3.0, and the Functional lncRNA Database. LncRNAdb and Noncode v3.0 databases provide information on lncRNAs expressed in different species, from yeast to plants. There are certain databases that provide information about the cell or tissue specific lncRNAs; for example, lncRNAdb, CHIPBase, Noncode v3.0, DIANA-LncBase, and lncRNome. Only lncRNAdb and Noncode v3.0 provide information about the cellular localization of the lncRNAs. There are certain databases which provide information about lncRNAs and their association with diseases: Noncode v3.0, lncRNAdb, DIANA-lncBase, lncRNADisease, and lncRNome. Few databases provide information about the association of lncRNAs with other non-coding RNAs. For instance, databases like DIANA-LncBase, LNCipedia, the Functional lncRNA Database, and lncRNome give information about the association between miRNAs and lncRNAs.

### Differential expression of lncRNAs in spermatogenesis and male infertility

#### Microarray

Microarrays with probes specific to lncRNAs detection became available about a decade back. These include Arraystar Mouse Stringent and Affymetrix Genechip 2.0 ST series and LncRNA microarray, which are based on updated lncRNA genomic annotation resources such as NONCODE, ENCODE, RefSeq, UCSC Genes, NRED, and fRNAdb. These platforms allow direct measurement of the levels of known lncRNA candidates. Only three microarray studies have investigated lncRNAs in relation to spermatogenesis [99–101] (Table 2). Sun et al. (2013) conducted a microarray study on neonatal (6 days) and adult mouse testis (8 weeks old) and identified 8265 lncRNAs, of which 3025 showed differential expression [99]. On comparing the two groups, they found that 1062 lncRNAs were significantly down-regulated and 1963 were significantly up-regulated in the adult mouse testis as compared to the neonatal testis. Some of the male germ cell specific lncRNAs, such as Aldoart2, Speer5-ps1, and Speer9-ps1 were enriched in adult testis and some of the imprinted lncRNAs, such as H19, Meg3, Airn were down-regulated in adult testis. The study also identified that there is a correlation between the lncRNAs expression and epigenetic modification marks, H3K4me3 and H3K27me3 [99]. However, no microarray study has analysed differential lncRNA expression in humans.

**Table 2:** List of transcriptome studies in spermatogenesis

Cell type/Tissue	Technique	Organism	Reference
Pre-natal day testes (E12.5 and E15.5) and post-natal day testes PND 7, 14, 21 and adult testes	Microarray	Mouse	[100]
Post-natal day testes PND 7, 14, 17, 21 and 28	RNA sequencing	Mouse	[102]
6 day and 8 week old testes	Microarray	Mouse	[99]
Spermatogonia (type A and B), pre-leptotene, combined leptotene/zygotene, pachytene spermatocytes and round spermatids	RNA sequencing	Mouse	[103]
Sperm and round spermatids	Stand-specific RNA sequencing	Mouse	[104]
Germ cells at mitotic, meiotic and post-meiotic stages	Single cell RNA sequencing	Mouse	[105]
Testes, brain, kidney, heart and liver	Microarray	Mouse	[101]
Sperm	RNA sequencing	Human	[106]
Spermatogonia, spermatocyte and spermatids	RNA sequencing	Human	[107]
A_pale_ and A_dark_ spermatogonia, leptotene/zygotene, early pachytene and late pachytene spermatocyte and round spermatids	RNA sequencing	Human	[108]
Testicular cells	RNA sequencing	Human	[109]
Motile and immotile (asthenozoospermic) sperm	RNA sequencing	Human	[110]
Sperm	RNA sequencing	Bovine	[111]
High-feed (HG) and hay testes	RNA sequencing	Sheep	[112]
High and low motility sperm	RNA sequencing	Bull	[113]

Bao et al. (2013) conducted another microarray study to identify lncRNAs expression at different developmental stages: embryonic day (E) 12.5, E 15.5, postnatal day 7 (PND 7), 14, 21, and adult [100]. They found that during fetal testicular development, 2593 lncRNAs were up-regulated and 1125 were down-regulated in E12.5 stage as compared to E15.5 stage. Further, 1976 lncRNAs were up-regulated whereas 3096 were down-regulated in P21 as compared to adult testes. In the other three time points, over 2000 and over 1500 lncRNAs were down- and up-regulated [100]. They selected 28 lncRNAs from microarray data for validation by real-time polymerase chain reaction (RT-PCR) and found a consistent expression of selected lncRNAs. These lncRNAs were AK005782, AK052477, BC049716, ENSMUST00000136860, ENSMUST00000145068, ENSMUST00000161511, ENSMUST00000172055, NR_027848, uc008hqc.1, uc009fxb.1, uc009rzf.1, AK020521, AK132382, ENSMUST0000098955, ENSMUST00000142338, ENSMUST00000153774, ENSMUST00000161823, NR_003270, NR_028123, uc009cxn.1, uc009ipj.1, uc009til.1, ENSMUST00000119676, ENSMUST00000131790, uc007kgi.1, uc007pgs.1, Tsx. In both the above studies, most of the differentially expressed lncRNAs overlapped with small RNAs, suggesting a possible precursor or regulatory relationship. Also, both these studies identified a close association between lncRNAs and epigenetic regulation. Bao et al. (2013) also identified by RNA immunoprecipitation experiment that 11 lncRNAs interact with one or many factors of chromatin-remodelling complexes, such as EZH2 and LSD1 [100].

In a recent study, microarray analysis of transcripts from mouse brain, kidney, heart, liver and testis was performed [101] (Table 2). It was found that the lncRNAs were most abundant in testes as compared to other tissues. A total of 14, 256 lncRNAs were detected in testis, out of which 1607 lncRNAs were specifically expressed in testis. They selected 26 testis-specific abundant lncRNAs for validation by reverse transcription polymerase chain reaction (RT-PCR) and found a pattern of expression similar to the microarray results. These testis-specific lncRNAs were AK018981, Uc007xmk.2, NR_01596, AK005929, NR_040424, ENSMUST00000086914, NR_102286, Uc008vyo.1, NR_028107, NR_027704, NR_038002, ENSMUST00000135865, NR_040326, Uc009dqq.1, NR_038180, ENSMUST00000131403, NR_045045, NR_033583, NR_003953, ENSMUST00000132787, NR_033788, NR_015571, NR_015547, ENSMUST00000136906, Uc029xya.1, and AK018904. The microarray studies on lncRNAs have been undertaken only on different developmental stages and between neonatal and adult testis to identify RNAs which participate in spermatogenesis. No study on purified germ cells or Sertoli cells has been undertaken. Studies on humans lack completely, providing a good opportunity to score the expression of lncRNAs between testicular tissues from azoospermic and normozoospermic individuals and in sperm from oligozoospermic infertile, normozoospermic fertile and normozoospermic infertile individuals.

#### RNA sequencing (RNA seq) studies

Because of the falling cost of the Next Generation Sequencing (NGS) technology, RNA seq has become a popular approach and is commonly used for transcriptome analysis. Its advantage over microarray technique is its sensitivity, coverage, resolution and the capability to identify new transcripts. Thousands of lncRNAs have been identified in human and mouse transcriptome sequencing projects [98]. RNA sequencing generates billions of reads having size in the range of 30–400 bp, depending upon the technology used. In RNA seq, the number of reads mapping to a region directly reflects the expression level of transcripts, facilitating comparison between two or more samples. Till date, several RNA sequencing studies have been conducted on testis or sperm in order to investigate their roles in male germ cell development. Nevertheless, due to huge data output from these studies, thousands of transcripts have been reported in relation to spermatogenesis.

##### Studies in humans

At least three studies have investigated lncRNAs in human germ cells (Table 2). Zhu et al. (2016) undertook sequencing and identified a total of 1800 lncRNAs, out of which 157 showed differential expression between different population of testicular cells [107]. In a similar study conducted by Jan et al. (2017) found a total of 137 lncRNAs were differentially expressed between different population of testicular cells [108]. Recently, Rolland et al. (2019) undertook RNA sequencing in human testicular cells to identify lncRNAs, and compared them with rat germ cell RNA profile [109]. They identified 113 known lncRNAs and 20 novel unannotated transcripts (NUTs) were conserved between human and rodents, suggesting them to be important for spermatogenesis. We compared the data across the above three studies and found 15 lncRNAs (LINC00635, LINC00521, LINC00174, LINC00654, LINC00710, LINC00226, LINC00326, LINC00494, LINC00535, LINC00616, LINC00662, LINC00668, LINC00467, LINC00608, LINC00658) to be common across them. We predicted their potential targets using lncRIsearch database, and found some crucial spermatogenic genes such as CENPB, FAM98B, GOLGA6 family, RPGR, TPM2, GNB5 and KCNQ10T1 (Table 3). In yet another search for targets using disease-specific target database (lncTarD), we identified TAZ, LIN28A, CDKN2B, CDKN2A, CDKN1A, CDKN1B, CDKN1C, EZH2, SUZ12, VEGFA as targets relevant to spermatogenesis for some of the lncRNAs (Table 3). This opens up new avenues of research in establishing regulatory connections to the signalling involved in spermatogenesis.

**Table 3:** Predicted and experimentally validated targets of selected spermatogenesis-associated lncRNAs

lncRNA gene ID	Global targets	Functions	Disease specific targets	Reference
LINC00635	**CENPB**^**a**^, **FAM98B**, **GOLGA6L1, GOLGA6L22, GOLGA6L6**, MLYCD, MUC2, RP11-573D15.8, **RPGR**, SNX8	Cenpb−/− male mice were fertile but have low sperm content and testis weight. FAM98A, **FAM98B** along with DDX1 and C14orf166 are required for PRMT1 expression (which is important for spermatogenesis). The whole **GOLGA6** family is highly expressed in testis. Overexpression of Rpgr in transgenic mice has led to increased flagellar defects and reduced sperm count.	–	[114–117]
LINC00174	RP11-573D15.8, CTD-2095E4.4, **GOLGA6L22, GOLGA6L6, RPGR**, SNX8	–	TAZ-involved in cancer progression.	–
LINC00654	ABI2, **FAM98B, GOLGA6L22, GOLGA6L6**, RP11-573D15.8, RP3-323A16.1, **RPGR**, SNX8	–	–	–
LINC00710	ABI2, CCDC85C, **FAM98B**, RP11-573D15.8, **RPGR**, SNX8	–	–	–
LINC00226	CCDC85C, **FAM98B**, SNX8, **TPM2**, C00717C00720L.1	**TPM2** was found to be up-regulated in cauda region as compared to caput/corpus region of human epididymis.	**–**	[118]
LINC00326	C9orf152, FLJ35934, **GNB5**, LINC00940,	**GNB5** could play a role in acrosome biogenesis and vesicle trafficking.	**–**	[119]
LINC00494	ABI2, EMC10, **FAM98B, GOLGA6L22, GOLGA6L6**, IGFN1, RP11-573D15.8, **RPGR**, SNX8	–	–	–
LINC00535	ABI2, **FAM98B**, SNX8	–	–	–
LINC00616	ABI2, CCDC85C, EMC10, **FAM98B**, MLYCD, PARVG, RP11-573D15.8, RP3-323A16.1, **RPGR**, SNX8	–	–	–
LINC00662	RP11-573D15.8	–	LIN28A- involved in cell metastasis and stemness in lung cancer.	–
LINC00668	ABI2, EMC10, **KCNQ1OT1**, MIR6820, RP11-573D15.8, RP3-323A16.1, **RPGR**, SNX8	KCNQ1OT1 is paternally expressed and plays a role in silencing of six genes *Ascl2, Kcnq1, Cdkn1c, Slc22a18, Phlda2,* and *Osbpl5.*	CDKN2B, CDKN2A, CDKN1A, CDKN1B, CDKN1C, EZH2, SUZ12, VEGFA- involved in cancer progression and cell proliferation.	[120]
LINC00467	RP3-323A16.1, **RPGR**	–	–	–
LINC00608	ABI2, EMC10, **FAM98B**, RP11-573D15.8, RP3-323A16.1, **RPGR**, SNX8	–	–	–
LINC00658	ABI2, AP006621.9, B3GALNT2, CCDC85C, **FAM98B**, RP11-573D15.8, **RPGR**, SNX8	–	–	–

^a^gene names in bold have known functions in spermatogenesis

LncRNA profiling of human sperm is very limited (Table 2). Sendler et al. (2013) sequenced sperm transcriptome of fertile men and identified 155 lncRNAs in human sperm [106]. Most of these lncRNAs were antisense or lincRNAs. Only one study analysed lncRNA profile in infertility. Zhang et al. (2019) undertook sequencing to identify lncRNAs that differ in motile and immotile human sperm [110]. While 9879 lncRNA genes (13,819 lncRNA transcripts) showed differential expression in between motile and immotile sperm, three lncRNAs i.e. lnc32058, lnc09522, and lnc98497 showed specific and high expression in immotile sperm in comparison to normal motile sperm. Gene ontology of lncRNAs targets revealed that most of the targets were involved in the process of spermatogenesis and sperm function.

##### Studies in mouse

Most of the RNA seq studies have been undertaken on mouse testis or sperm (Table 2). Laiho et al. (2013) undertook RNA sequencing on whole testis of mouse on post-natal days 7, 14, 17, 21, and 28 [102]. When compared between two adjacent developmental stages, they found that 17–46 lncRNAs were down-regulated and 16–605 lncRNAs were up-regulated in each transition. Wichman et al. (2017) analysed the expression of lncRNAs at specific stages of spermatogenesis in mouse by purifying key stages (combined type A and type B spermatogonia, pre-leptotene, combined leptotene/zygotene, pachytene spermatocytes and round spermatids) of spermatogenesis by STA-PUT method [103]. They found that 1630 lncRNAs were differentially expressed in spermatogonia versus pachytene spermatocytes with Gm14244, 1700026D11Rik, 1700066O22Rik, RP24-503F14.1, Gm1698, 1700110I01Rik, Gm20621, Gm10619, RP23-52 K8.2, 1700006J14Rik, H19, Gm26751, Gm13054, Gm26656, Gm26835, 5930412G12Rik, Gm26775, D830015G02Rik, Gm26673, Gm5532 being the top 20. 1534 lncRNAs were differentially expressed between pachytene spermatocytes and round spermatids with 1810059H22Rik, 4930412B13Rik, 4930467K11Rik, Gm14249, Astx6, RP23-245G9.1, Gm11782, Gm13481, 9130204K15Rik, Gm13175, Gm27032, Gm16278, 1700040D17Rik, Gm10619, Gm14244, 4930477E14Rik, RP23-46B12.8, 1520401A03Rik, A730090N16Rik, Gm13589 being the top 20. They also identified a subset of lncRNAs that escape meiotic sex chromosome inactivation at the pachytene stage of meiosis and others with strong testicular expression pattern, suggesting their importance in spermatogenesis. Lastly, they generated a knock out mouse having the deletion of one X-linked lncRNA, Tslrn1 (testis-specific long noncoding RNA1) and found that males carrying this deletion displayed normal fertility, but a significant reduction in sperm count [103]. This shows its participation in the regulation of spermatogenesis, though the molecular mechanisms of its action remain to be investigated.

Zhang et al. (2017) undertook strand-specific RNA sequencing that revealed a large repertoire of lncRNAs in mouse mature sperm [104]. They identified 20,907 known and 4088 novel lncRNAs transcripts in sperm. Chen et al. (2018) undertook single-cell-RNA sequencing on mouse germ cells at mitotic, meiotic and post-meiotic stages in order to identify the molecular events occurring during male germ cell development and to reveal several novel crucial regulators of mammalian spermatogenesis [105]. A large proportion of known protein-coding genes (18,037 out of 20,088, 89.8%) were identified in the spermatogenic cells. They also identified 9431 out of 11,962 (78.8%) annotated lncRNAs to be expressed in the spermatogenic cells, which had different spatio-temporal expression. The highest numbers and the expression level of lncRNAs were found in the diplotene, MI spermatocytes and steps 6–8 spermatids.

##### Studies in other animals

Selvaraju et al. (2017) undertook sequencing to identify the spermatozoal transcripts in fresh bull semen [111] (Table 2). They found that spermatozoa retain a variety of RNAs, including lncRNAs. The most abundant lncRNAs reported in this study were NONBTAT002138.2, NONBTAT029740.1, NONBTAT026069.2, NONBTAT026075.2, NONBTAT015718.2, NONBTAT015717.2, NONBTAT017665.2, NONBTAT027794.1, NONBTAT007060.2, NONBTAT029554.1, NONBTAT008960.2, NONBTAT022624.2, NONBTAT030080.1, NONBTAT030770.1, NONBTAT030342.1, NONBTAT030589.1, NONBTAT031209.1, NONBTAT022014.2, NONBTAT025041.2, and NONBTAT001981.2. It remains to be seen if some of these lncRNAs match with human or rodent sperm.

Yanli et al. (2017) conducted RNA sequencing to identify lncRNAs and their role in the testes development of high-grain (HG) diet fed sheep [112]. A total of 6460 lncRNAs were reported. Further, on comparing the lncRNAs expressed in testes of hay and HG diet groups, researchers identified 28 up-regulated and 31 down-regulated lncRNAs differentially expressed between the two groups. Ten differentially expressed lncRNAs were randomly selected to validate the RNA sequencing results by using qRT-PCR. These lncRNAs were XR_001435329.1, XR_001025228.2, XR_0010443569.1, XR_001042765.2, XR_001045031.2, XR_001040775.1, XR_001434819.1, XR_001044933.2, XR_001044935.2, XR_001044859.2. This study apart from providing lncRNA profile of sheep testis also suggested a significant role of 59 lncRNAs in regulating the effect of nutrition on fertility.

In another study, Wang et al. (2019) undertook an integrated analysis of lncRNAs and mRNAs in high and low motility Holstein bull sperm, in order to identify mRNAs regulated by lncRNAs and their impact on sperm motility [113] (Table 2). They reported 11,561 lncRNAs in sperm, of which 2517 were differentially expressed between high and low motility sperm groups. The top 20 significantly differentially expressed lncRNAs reported were TCONS_00000538,TCONS_00003123, TCONS_00005494, TCONS_00007217, TCONS_00008747, TCONS_00009226, TCONS_00011237, TCONS_00012048, TCONS_00012181, TCONS_00012775, TCONS_00013706, TCONS_00014744, TCONS_00014816, TCONS_00015123, TCONS_00019053, TCONS_00020130, TCONS_00021031, TCONS_00021657, TCONS_00024090 and TCONS_00025506. They also found that out of the 20,875 protein coding genes detected in semen, 19 were differentially expressed between high and low motility groups. Further, they found that lncRNA TCONS_000417333 targets a gene EFNA1 (ephrin A1), which is involved in male reproductive physiology. Several other lncRNAs and mRNAs identified in this study are good targets for further studies. This is a landmark study in identifying bull sperm motility and fertility lncRNA markers, which can find potential application in animal husbandry.

### Functional lncRNAs in spermatogenesis

**Fig. 4:**
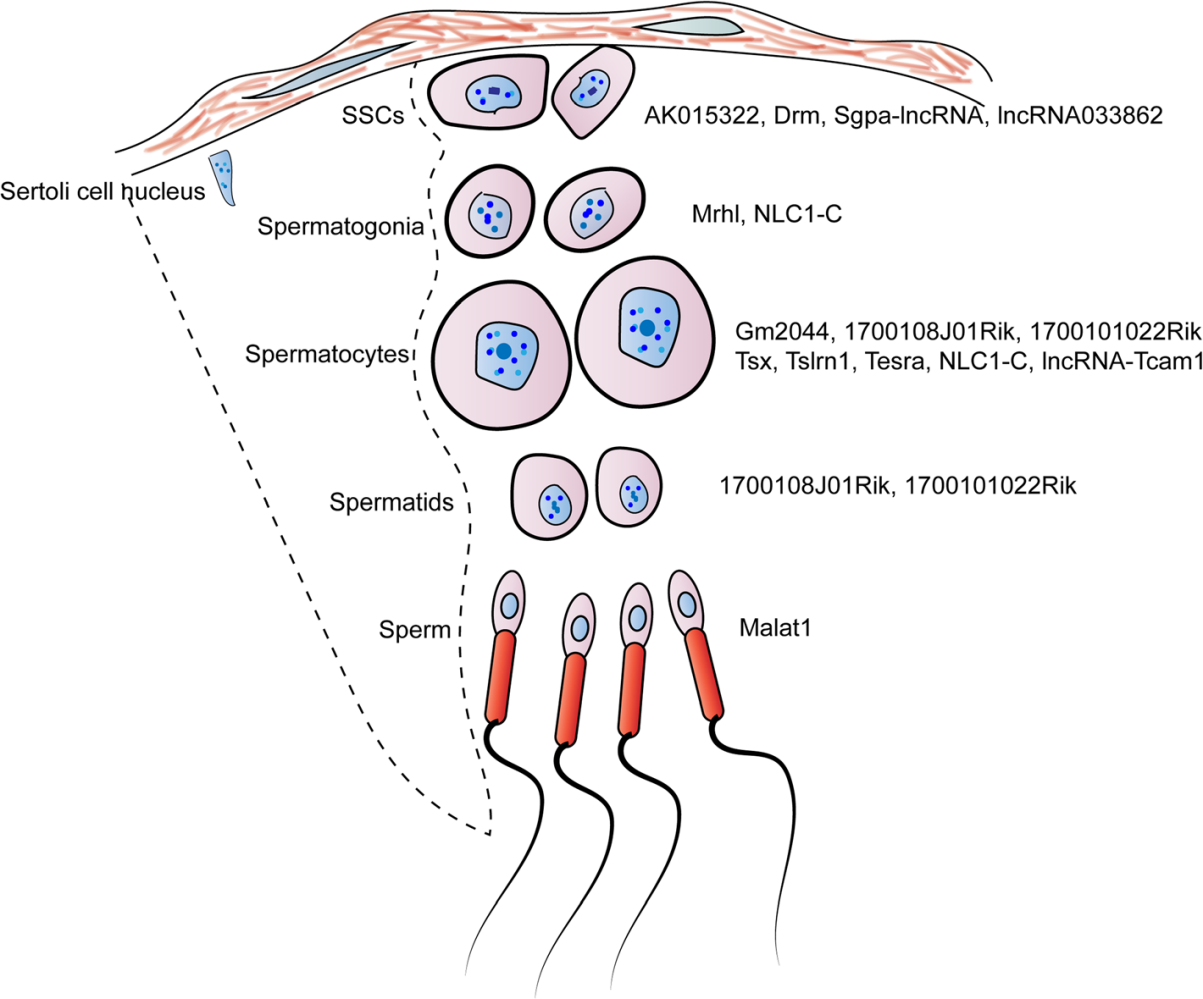
Functionally characterized lncRNAs expressed in different stages of spermatogenesis

Many potential lncRNAs have been identified in male germ cell development, some of them showing differential expression between fertile and infertile sperm. However, only a few have been functionally annotated and characterized [1] (Fig. 4). Below are some lncRNAs, which have been shown to participate in spermatogenesis.

#### Humans

##### Narcolepsy candidate-region 1 gene (NLC1-C)

Narcolepsy candidate-region 1 gene (NLC1-C) is an lncRNA expressing in the cytoplasm of spermatogonia and early spermatocytes. It was found that the over-expression of NLC1-C promoted cell growth whereas its loss inhibited cell growth and promoted apoptosis [121]. Microarray analysis revealed that NLC1-C had lower expression in MA (maturation arrest) patients than those with normal spermatogenesis. In another study, it was reported that NLC1-C inhibited the transcription of miR-320a and miR-383 by binding to the RNA-binding domain of nucleolin and promoted the proliferation of spermatogonia and spermatocytes in MA patients [121].

#### Rodents

##### Meiotic recombination hot spot locus (Mrhl)

Meiotic recombination hot spot locus (Mrhl) RNA is a 2.4 monoexonic residing in the nucleus. Mrhl was first identified by Nishant et al. (2004) while working on a 17.2 kb fragment of a novel meiotic recombination hot spot in mouse chromosome 8 [122]. Later, the same research group showed that Mrhl RNA was cleaved by Drosha to form an 80-nucleotide RNA intermediate [123]. Further evidence showed that both 2.4 kb full length and cleaved 80 nucleotide RNAs were localized in the nucleoli of GC1 cell line, suggesting their possible role in some specific chromatin domains [123]. Further studies by the same research group showed that Mrhl interacts with p68 and inhibits the Wnt signaling pathway [124]. Recently, they identified that the transcription factor Sox8 is a regulatory link between mrhl RNA expression, Wnt signaling activation and meiotic progression. In this study, Mrhl was shown to regulate Sox8 by binding to its promoter. Mrhl RNA interacts with other regulatory factors like Myc-Max-Mad, co-repressor Sin3a and co-activator Pcaf at the Sox8 promoter site and regulates spermatogonial differentiation [124].

##### HongrES2

HongrES2 is a 1588 bp lncRNA, which is co-transcribed by chromosomes 5 and 9 in rats. Its expression is seen in the cauda region of epididymis [125]. The expression of HongrES2 was found to increase constantly till the first wave of spermatogenesis and become constant around day 450 in rats. This expression pattern of hongrES2 perfectly matches with its reported role in regulating sperm maturation. In the nucleus, HongrES2 is processed to a 23 bp RNA called as mil-HongrES2. This processed transcript inhibits the expression of CES7, an epididymis specific protein. Overexpression of mil-HongrES2 resulted in decreased sperm capacitation [125], indicating that low HongrES2 expression promotes sperm maturation process in the epididymis.

##### Testis-specific X-linked (Tsx)

Testis specific X-linked (Tsx) is an lncRNA, which is located within the X-inactivation center. It is known to be highly expressed in the pachytene spermatocytes and not in spermatogonia or round spermatids, suggesting its role in regulating the meiotic division. An increased number of abnormal pachytene spermatocytes undergoing apoptosis were seen in Tsx knockout mice, suggesting its role in the progression of meiosis in mice [126].

##### Dmrt1-related gene (Drm)

Similarly, Dmrt1-related gene (Drm) is an exclusively expressing lncRNA in testis. It was discovered by Zhang et al. (2010) in an attempt to clone the Dmrt1 gene when they found another RNA isoform of different size and sequence on the 3’half [127]. Later, it was found that this 3′ half was encoded by a gene present on chromosome 5, whereas Dmrt1 gene is present on chromosome 19. This chimeric form of mRNA was the result of disrupted coding region of Dmrt1 gene and the replacement of its 3’UTR region, which resulted in decreased expression of normal Dmrt1 protein. Dmrt1 is known to play a crucial role in spermatogonial development by upregulating *Sohlh1* and prevents early meiosis of spermatogonia by repressing Stra8 [128, 129]. Therefore, the regulation of Dmrt1 by Drm could be involved in the switching between mitosis and meiosis during germ cell development.

##### Spga-lncRNAs

Chan et al. (2006) used two different techniques, SAGE and whole genome tiling microarray, in three different germ cell stages: type A spermatogonia, pachytene spermatocytes, and round spermatids to identify the protein coding genes [130]. Later, Lee et al. (2012) used this published SAGE and tiling microarray data to identify lncRNAs [131]. With this approach, they found 50, 35, and 24 single-exonic lncRNAs with expression in type A spermatogonia, pachytene spermatocyte and round spermatids, respectively. After validation by qPCR, northern blotting and RACE, two lncRNAs Spga-lncRNA1 and Spga-lncRNA2 were found to be highly expressed in spermatogonia. Further, in vitro experiments showed that they may play important roles in maintaining stemness of spermatogonia.

##### LncRNA- testicular cell adhesion molecule 1 (lncRNA-Tcam1)

LncRNA- testicular cell adhesion molecule 1 (lncRNA-Tcam1) is a 2.4 kb nucleotide long nuclear lncRNA. It is exclusively expressed in mouse male germ cells and is assumed to play a crucial role in meiosis. In a recent report, it was found that lncRNA-Tcam1 regulates immune related genes in mouse germ cells [132]. The induction of lncRNA-tcam1 transcription using the tet-off system in GC-2 cells upregulated six genes, Trim30a, Ifit3, Tgtp2, Ifi47, Oas1g, and Gbp3. All these genes were found to be associated with immune response, suggesting that lncRNA-Tcam1 regulates immune related genes.

##### Tug1 lncRNA

Tug1 locus resides on chromosome 11 in murine and is among the most conserved lncRNAs between mouse and humans [133]. The expression of this lncRNA was seen not only in adult tissues but also in a number of embryonic tissues during the embryonic development in both human and mouse. Lewandowski et al. (2019) reported that the loss of Tug1 locus leads to male sterility [133]. They found a significant decrease in sperm number in Tug1^−/−^ males as compared to the wild-type mice (oligospermia), they also found morphological defects in the head and mid-piece region of sperm (teratospermia). Thus, these findings revealed an essential role of the Tug1 locus in male fertility. Further investigations showed that Tug1 locus harbours three distinct regulatory activities: cis-acting repressive DNA function, trans- acting Tug1 lncRNA and peptide (TUG1-BOAT) having role in maintaining mitochondrial membrane potential [133].

##### Tesra

Tesra is a 4435 nucloetide long testis-specific lncRNA, which is present at the *Prss/Tessp* gene cluster on chromosome 9 [134]. The expression of tesra was detected in the cytoplasm, and the nucleus of germ cells and only in the cytoplasm of Leydig cells while no expression was seen in the Sertoli cells. Satoh et al. (2019) reported a nuclear function of this lncRNA where it acts as a transcriptional activator of the *Prss/Tessp* gene. The *Prss/Tessp* gene cluster specifically activates in the primary spermatocytes at the late pachytene stage and has been found to be critical for meiotic progression of the germ cells. The molecular mechanism by which tesra enhances the activity of *Prss/Tessp* gene promoter still needs to be explored [134]. However, previous studies have shown nuclear lncRNAs to recruit histone modifiers or transcription factors to chromatin regions of target genes or work as a scaffold that interacts with multiple protein complexes to regulate the target gene [134].

##### LncRNA033862

This lncRNA resides on chromosome 19 and is transcribed from the antisense strand of the GDNF family receptor alpha (*Gfra1*) gene. LncRNA033862 was found to be highly dysregulated in response to GDNF (a ligand of GFRA1) in mouse SSCs [135]. GFRA1 has been considered as a cell surface marker of SSCs and the loss of Gfra1 has been reported to disrupt SSC self-renewal in neonatal mouse testis. It was found that this lncRNA is predominantly expressed in spermatogonia and SSCs and regulates SSC self-renewal by acting as a transcriptional activator of Gfra1 [135].

##### AK015322

Hu et al. (2016) performed a microarray study to investigate the expression profile of lncRNA in different germ cell populations (spermatogonial stem cell, type A spermatogonia, pachytene spermatocyte and round spermatids) [136]. They found lncRNA AK015322 expression to be highest in the spermatogonial stem cells as compared to other germ cells. Knockdown of AK015322 resulted in decreased proliferation, suggesting its role in maintaining SSC self-renewal capacity. AK015322 regulates the expression of ETV5, important for SSC proliferation, by working as a decoy of miR-19b-3p [136]. Therefore, this study revealed that lncRNA AK01522 suppresses the effect of miR-19b-3p on ETV5 expression, thus promoting the proliferation of SSCs.

##### Gm2044

In a microarray study, the expression of Gm2044 was found to be highest in the pachytene spermatocytes of mouse testis [137]. Further, the expression of Gm2044 was found to be more at day 12 to day 20, which represents spermatocyte formation and meiotic phase during the first wave of spermatogenesis, suggesting its role in meiotic progression. RNA pull down experiment revealed that Gm2044 interacts with Utf1 and the overexpression of Gm2044 resulted in decreased protein level of UTF1, suggesting Utf1 to be a direct target of Gm2044. The study identified Gm2044 to be critical for germ cell transition and meiotic progression by inhibiting Utf1 translation [137].

## Conclusion and future perspective

Once thought to be junk, non-genic regions of DNA encode several non-coding RNAs, which play important roles in gene regulation. The advent of next generation sequencing has accelerated the discovery of lncRNA along with other types of non-coding RNAs. Investigations on lncRNAs expression in the embryonic and post-natal testis identified about 1000 lncRNAs that show change in expression with the onset and progression of spermatogenesis. At least one study investigated lncRNAs in different germ cells in adult testis and identified about 1500 such RNAs showing change in expression during spermatogenesis. Specific investigations on particular lncRNAs identified their impact on sperm count (Tslrn1, Tug1), sperm motility (lnc32058, lnc09522, and lnc98487), sperm capacitation (mil-hongrES2) and meiosis (Drm, Tsx, Gm2044). There are certain lncRNAs involved in maintaining the balance between stemness of spermatogonia and their differentiation. For example, Spga-lncRNA, AK015322, LncRNA033862 are involved in maintaining the stemness of spermatogonia while Mrhl lncRNA binds to the Sox8 promoter to regulate spermatogonial differentiation in mouse. Comparison across three studies on human germ cells identified 15 (LINC00635, LINC00521, LINC00174, LINC00654, LINC00710, LINC00226, LINC00326, LINC00494, LINC00535, LINC00616, LINC00662, LINC00668, LINC00467, LINC00608, LINC00658) common lncRNAs that can be great candidates for further analysis for their role in spermatogenesis and their differential expression in infertility.

A number of databases and analysis tools have now become available to facilitate the data analysis and comparison. Transcriptome sequencing offers the identification of novel RNAs along with known RNAs; therefore, sequencing studies should provide new candidate RNAs for further investigation in spermatogenesis and male infertility. Several lncRNAs have been identified which play roles in different developmental stages and adult mouse testes. Hong et al. (2018) identified testis-specific lncRNAs in mouse, of which 26 most abundant(AK018981, Uc007xmk.2, NR_01596, AK005929,NR_040424, ENSMUST00000086914, NR_102286, Uc008vyo.1, NR_028107, NR_027704, NR_038002, ENSMUST00000135865, NR_040326, Uc009dqq.1, NR_038180, ENSMUST00000131403, NR_045045, NR_033583, NR_003953, ENSMUST00000132787, NR_033788, NR_015571, NR_015547, ENSMUST00000136906, Uc029xya.1, AK018904) are strong candidates for further investigations. Bao et al. (2013) reported 28 candidate lncRNAs (AK005782, AK052477, BC049716, ENSMUST00000136860, ENSMUST00000145068, ENSMUST00000161511, ENSMUST00000172055, NR_027848, uc008hqc.1, uc009fxb.1, uc009rzf.1, AK020521, AK132382, ENSMUST0000098955, ENSMUST00000142338, ENSMUST00000153774, ENSMUST00000161823, NR_003270, NR_028123, uc009cxn.1, uc009ipj.1, uc009til.1, ENSMUST00000119676, ENSMUST00000131790, uc007kgi.1, uc007pgs.1,Tsx) which were differentially expressed across different developmental stages in mouse.

Although many potential lncRNAs have been discovered till date with the help of advanced technologies and public lncRNA annotation resources, yet the functional annotation of lncRNAs in spermatogenesis is in infancy and has tremendous possibilities. Long noncoding RNAs involved in spermatogenesis can be identified by using public lncRNA annotation, but their specific localization and biological function in testis need experimental evidence. A number of studies have undertaken RNA sequencing in testis, germ cells and sperm [108, 138–140]. Some of these have compared RNA sequencing data between fertile and infertile men. However, most of these studies did not pay attention to lncRNA. One of the reasons could be the lack of tools available to analyse lncRNAs. The availability of such tools now provides an opportunity to analyse their transcriptome data for the analysis of lncRNAs. LncRNAs may be used as biomarkers or/and targets to diagnose or/and treat infertility in the future when more high throughput data, more refined bioinformatics tools, protein-RNA binding models, and regulatory evidence from ENCODE become available.

New techniques have been developed to identify the function of lncRNAs through their interaction with other molecular species. These techniques involve the identification of interacting partners like RNA, protein and/or DNA and isolation of a component of interest (RNA/protein). For identification of RNAs linked to proteins, RIP (RNA immunoprecipitation) and HITS/PAR-CLIP (High-Throughput Sequencing of RNA/PhotoActivatable-Ribonucleoside-Cross Linking and Immunoprecipitation) techniques can be used. Other techniques like RNA pull down and/or ChIRP/CHART would be useful to identify proteins and DNA sequences associated with a particular lncRNA.

## Data Availability

No associated data is available.
